# Hope and Optimism in 2026

**DOI:** 10.31486/toj.25.5058

**Published:** 2025

**Authors:** Ronald G. Amedee

**Affiliations:** Director of Clinical School and Professor, The University of Queensland Medical School, Ochsner Clinical School, New Orleans, LA; Editor-in-Chief, *Ochsner Journal*

*Learn from yesterday, live for today, hope for tomorrow.*

–*Albert Einstein*

The Winter 2025 issue of *Ochsner Journal* contains three original research articles, one letter to the editor, one editorial, one innovative program article, one review and contemporary update article, and four case reports and clinical observations. The letter to the editor, from Ochsner Health physicians in radiology and neurosciences, details “Reading Between the Striatal Lines: Magnetic Resonance Imaging Insights Into Huntington Disease.” The editorial, “Family Connects New Orleans: Redefining Fourth Trimester Care,” is by Ochsner Health professionals who work with new moms and babies in the postpartum Family Connects program.

Our first original research article comes from Ochsner Otolaryngology in New Orleans and Shreveport. Kattar, Levy, and McCoul examine the “Short-Term Risk of Complications Related to Obstructive Sleep Apnea After Sinonasal Surgery,” but instead of limiting their analysis solely to sinonasal surgery, the authors provide data on complications after 6 different kinds of surgery in patients with and without obstructive sleep apnea. For all surgery types, patients with sleep apnea had a greater incidence of supplemental oxygen requirement. The second original research article, a collaboration between Ochsner and Tulane, is an update of allergen sensitization patterns in the New Orleans area. The researchers wanted to determine if the patterns had changed since the update that was done shortly after Hurricane Katrina. Along with the expected results (high prevalence to sensitization to dust mites and other common allergens), “Examination of Aeroallergen Sensitization Patterns in Southeastern Louisiana” found a surprising result: a high prevalence of *Acremonium* sensitization and very low sensitization to other fungi. The final original research article in this section is also Ochsner work. Three clinical pharmacists—Alyssa Stutes, Steven Quoc Thai, and Brooke Baetz—worked with cardiologist Dr Selim Krim and biostatistician Cruz Velasco-Gonzalez on “Effect of Statin Potency on Rapid Coronary Intimal Thickening and Rejection in Heart Transplant Recipients.” Their findings show that increased statin doses for patients after heart transplant were safe and seemed to reduce rejection but did not attenuate cardiac allograft vasculopathy at 1 year.

The innovative program article is from staff members in the Ochsner Clinical Simulation and Patient Safety Center. “Use of a Novel Training Aid for Teaching the Nursing Care of Central Venous Catheters” provides illustrated step-by-step instructions for creating a reusable central line dressing for hands-on training. With low-cost materials and ingenuity, the sim center staff eliminated the cost of using a new dressing for each trainee.

Under the direction of Ochsner anesthesiologist Dr Bobby Nossaman, two University of Queensland-Ochsner Clinical School students authored the review “Pharmacologic Management of Central Fever in Patients With Acute Brain Injury.”

All but one of the case reports and clinical observations included in the winter issue have Ochsner authors. “Treatment of Spinal Epidural Abscess With Limited Decompression” is by Ochsner-affiliated authors Alani, Taylor, and Yu. University of Pittsburg and Duke University colleagues Raza and Kocasarac coauthored “Cobalt Toxicity Presenting as Bilateral Optic Neuropathy.” “The Potential Role of Zilretta in Reducing Intra-Articular Effusions” is another Ochsner case report, and two authors of “Esophageal Cancer in a Patient With Poland Syndrome” are Ochsner staff members.

This edition of the *Journal* will be published as we approach the holiday season and year's end. This is the perfect time to thank all our reviewers for their efforts and also to thank all the authors who submitted their work for review, and in some cases, the successful publication of their articles during calendar year 2025. I also wish to thank Kathleen McFadden for her awesome contributions as manager-medical editor of the *Journal*. Her diligence to task makes all of our publishing efforts possible. As we close out 2025, I particularly like the wisdom offered by Einstein that might serve as a valuable preparation for New Year 2026. May we all learn from the past, live for the current moment, and enter the future year with enduring hope and optimism. Happy Holidays to all!

